# Beyond facial injury: prognostic utility of maxillofacial trauma scores in the emergency department

**DOI:** 10.1007/s00068-026-03143-2

**Published:** 2026-03-06

**Authors:** Elif Kamış Boztepe, Bedriye Müge Sönmez, Gülşen Akçay

**Affiliations:** 1 Department of Emergency Medicine, University of Health Sciences, Dışkapı Yıldırım Beyazıt Research and Training Hospital, Zübeyde Hanım, Şht. Ömer Halisdemir Blv No:1, Altındağ, Ankara, 06110 Turkey; 2 Present Address: Department of Emergency Medicine, Eyüp Sultan State Hospital, İstanbul, Turkey; 3 Present Address: Faculty of Medicine, Department of Emergency Medicine, Dokuz Eylül University, İzmir, Turkey; 4 Present Address: Department of Emergency Medicine, Etlik City Hospital, Ankara, Turkey

**Keywords:** Maxillofacial trauma, MFISS, FISS, Emergency department

## Abstract

**Purpose:**

To evaluate the prognostic utility of two widely used maxillofacial trauma scoring systems (MFTSs) -the Facial Injury Severity Score (FISS) and Maxillofacial Injury Severity Score (MFISS)- in adult multiple trauma patients in the emergency department (ED).

**Methods:**

This prospective observational study was conducted in the ED of a tertiary care hospital between 01.08.2020 and 01.08.2021. A total of 124 MFT patients aged ≥ 18 years with at least one concomitant injury to another organ system were included. FISS and MFISS were calculated for each patient. The associations between scores and coexisting injuries, intensive care unit (ICU) admission, and mortality were analyzed. Predictive performance for mortality was assessed using receiver operating characteristic (ROC) curve analysis.

**Results:**

Higher FISS scores were significantly associated with intracranial injuries, including subarachnoid hemorrhage (SAH), cranial fractures, and pneumocephalus (all *p* < 0.01), as well as with pneumothorax, rib fractures, ICU admission, and mortality (all *p* ≤ 0.004). MFISS scores were significantly higher in patients with SAH, while a negative association was observed between MFISS and abdominal injuries (*p* = 0.043). ROC analysis demonstrated good predictive performance for mortality, with areas under the curve of 0.820 for FISS and 0.758 for MFISS (both *p* < 0.01).

**Conclusions:**

FISS and MFISS are associated with clinically meaningful outcomes and may serve as practical tools for early risk stratification and prognostic assessment in the ED among adult multiple trauma patients with maxillofacial injuries.

## Introduction

Maxillofacial trauma (MFT) in the emergency department (ED) is frequently associated with life-threatening systemic injuries and represents a significant clinical challenge beyond facial deformity alone [1–3]. This suggests that MFT could be a clinical indicator of trauma patients at high risk, as it may pose a potential risk for airway management- one of the cornerstones of ED trauma care- highlighting the importance of valid ED-based severity assessment instruments [1, 2]. There are a number of maxillofacial trauma scoring systems available, but their primary purpose is not to aid rapid emergency decision-making but rather to predict factors such as surgical complexity, treatment costs, and hospital stays [4–7].

Beyond characterizing the extent of the facial injury, the clinical utility of maxillofacial trauma scoring in the ED depends on whether these scores can represent the patient’s total risk profile in the acute setting [1–3]. Here, two distinct yet complementary severity frameworks with different clinical implications, the Facial Injury Severity Score (FISS) and the Maxillofacial Injury Severity Score (MFISS), may help categorize patients at risk and determine their prognosis following MFT [4, 5]. While FISS primarily quantifies fracture burden and anatomical distribution, which may serve as a surrogate marker of trauma energy transfer and signal an increased likelihood of associated intracranial or systemic injuries [3, 8], MFISS takes into account functional impairment and anatomical severity according to the Abbreviated Injury Scale (AIS), which may indicate physiological compromise, injury complexity, and overall clinical vulnerability [5–7]. Accordingly, comparing FISS and MFISS in the ED setting allows evaluation of whether a fracture-oriented or a function-oriented severity approach more closely aligns with clinically meaningful risk in patients presenting with MFT and concomitant injuries. In other words, the question is not merely ‘how broken is the face?’ but ‘how threatened is the patient?’ in the ED.

Given the severe and potentially life-threatening complications associated with MFTs, there is a gap in ED triage regarding the applicability of maxillofacial trauma scoring systems (MFTSs) to polytrauma patients. Therefore, this study aims to compare the prognostic utility of different MFTSs for ED acute decision-making and to evaluate their clinical relevance beyond prior research that focused on injury severity or cost-related measures rather than injury outcomes.

## Methods

### Study design and setting

This prospective observational study was conducted in multiple trauma patients aged ≥ 18 years who consecutively presented to the ED of a tertiary care hospital with MFT between 01.08.2020 and 01.08.2021. The study was approved by the Institutional Ethics Committee (Decision Number: 91/18). Written informed consent was obtained from all patients or their first-degree relatives who were unable to provide consent prior to the procedures, in accordance with the Helsinki Declaration (2013). Patient confidentiality was ensured by restricting access to study data to authorized personnel only.

### Sample size and study population

A priori sample size calculation was performed using G*Power software, which indicated that a minimum of 122 patients would be required to achieve 95% statistical power with a type I error rate of 5%. During the study period, 10,000 patients presented to the ED; among them, all consecutive adult patients with MFT and at least one concomitant injury involving another organ system were screened and enrolled until the required sample size was reached. After reaching the target sample size, no further eligible patients were included. Patients were included if they were aged ≥ 18 years, presented with MFT, and had at least one concomitant injury involving another organ system. Patients younger than 18 years, pregnant patients, patients with isolated MFT, patients referred from or transferred to another healthcare facility, and patients who declined to participate were excluded from the study.

### Data collection

Demographic characteristics, mechanism of injury, injured anatomical regions and organ systems, imaging modalities performed, therapeutic interventions, ED and clinical outcomes, length of hospital stay (LOS) and/or ICU stay, and calculated MFTSs were recorded using a standardized data collection form. The location and anatomical area of MFTs were classified according to the maxillofacial anatomic sites evaluated in MFTSs. Mechanisms of injury were classified as assault, motor vehicle crash (MVC), pedestrian crash (PC), and falling from a height.

### MFTSs

FISS [4] and MFISS [5] were calculated for all included patients. These scoring systems were chosen to illustrate two viewpoints on the severity of MFT in the ED. Table 1 details the components, scoring steps, and calculation procedures for both scores. To summarize, FISS mainly analyzes the number of fractures and their anatomical distribution on the face, whereas MFISS assesses injury severity and functional impairment using the AIS.

**Table 1:** Individual scoring stages of the Facial Injury Severity Score (FISS) and the Maxillofacial Injury Severity Score (MFISS)

Scoring system	Anatomical region / component	Injury description	Score
FISS	Lower face (Mandible)	Dentoalveolar fracture	1
		Body / ramus / symphysis fracture (each)	2
		Condylar or coronoid fracture (each)	1
	Midface	Dentoalveolar fracture	1
		Le Fort I fracture*	2
		Le Fort II fracture*	4
		Le Fort III fracture*	6
		Naso-orbito-ethmoidal fracture	3
		Zygomaticomaxillary complex fracture	1
		Nasal bone fracture	1
	Upper face	Orbital rim or roof fracture	1
		Displaced frontal sinus or frontal bone fracture	5
		Non-displaced frontal fracture	1
	Soft tissue injury	Facial laceration > 10 cm	1
MFISS	AIS-based anatomical injury (A1–A3)	Skin/subcutaneous contusion or laceration < 25 cm²; oral mucosa or tongue injury; ramus or nasal fracture; dental fracture; temporomandibular joint contusion	1
		Laceration > 10 cm or soft tissue avulsion > 25 cm²; alveolar fracture; condylar fracture; mandibular body fracture; maxillary fracture (Le Fort I–II); displaced nasal fracture; orbital fracture; temporomandibular joint dislocation; zygomatic fracture; facial nerve branch injury	2
		Le Fort III fracture with < 20% blood loss; displaced comminuted orbital fracture	3
		Maxillary fracture with > 20% blood loss	4
	Limited mouth opening (LMO)	Mouth opening 2–3.7 cm	1
		Mouth opening < 2 cm	2
	Malocclusion (MO)	Unilateral malocclusion (< 6 teeth)	1
		Unilateral malocclusion (> 6 teeth)	2
		Bilateral malocclusion	3
	Facial deformity (FD)	Open soft tissue injury < 4 cm without tissue defect; non-displaced facial fracture	1
		Open soft tissue injury > 4 cm with tissue defect < 2 cm²; displaced fracture; bone defect < half of one jaw; facial nerve branch injury	2
		Open soft tissue injury > 4 cm with tissue defect > 2 cm²; bone defect > half of one jaw or bilateral jaw defect; facial nerve trunk injury	3

### Outcomes

The primary outcome of the study was the association of FISS and MFISS with clinically relevant outcomes in the ED and during hospitalization, including mortality, need for ICU admission, and LOS. Secondary outcomes included the predictive utility of these scoring systems for the presence of associated systemic injuries.

### Statistical analyses

Data were analysed using the Statistical Package for the Social Sciences^®^ (SPSS, IBM-Illinois, USA) for Windows Version 23.0. Normality of continuous variables was evaluated using the Kolmogorov-Smirnov test, along with skewness and kurtosis as descriptive statistics. All tests were performed nonparametrically because the data were not normally distributed in at least one group across all datasets. Descriptive statistics were given as the number of patients (n) and percent (%) in the representation of categorical variables, and the median (minimum-maximum) in the representation of numerical variables. A difference analysis of numerical variables between two independent groups was performed using the Mann-Whitney U test. Comparison of ratios between independent groups was performed using the Chi-square test. Spearman’s correlation analysis was used to assess the correlation between continuous variables. In cases where the tests affecting the data yielded dichotomous results, predictive analysis of mortality and morbidity for continuous variables and determination of the threshold value were performed using ROC analysis. Statistical alpha significance levels were set at *p* < 0.05.

## Results

### General characteristics and coexisting injury profile of the study group

During the study period, a total of 10,252 trauma patients were admitted to the ED. A total of 124 patients who met the inclusion criteria were included in the study. The median age of the patients was 41.5 (range: 18–92). The majority of patients were male (75.8%, *n* = 94). The most common mechanism of injury was MVC (*n* = 68, 54.8%). Among concomitant injuries, head trauma was the most common (64%) associated injury, whereas abdominal injury (20%) was the least. In this context, patients were grouped by body region, and, as a subgroup analysis, the least associated injuries were cervical and thoracic spine injuries (8.1% and 8.9%, respectively), followed by pelvis fracture (7.3%) and hemothorax (4%). The distribution of associated injuries was detailed in Table 2.

**Table 2:** Coexisting injuries of the study population

Injury category	*n* (%)
**Head injury**	64 (53.3)
Subdural hematoma	23 (18.5)
Epidural hematoma	22 (17.9)
Subarachnoid hemorrhage	16 (12.9)
Fracture (skull)	24 (19.4)
Pneumocephalus	42 (33.9)
**Spinal injury**	35 (28.2)
Cervical vertebra fracture	10 (8.1)
Thoracic vertebra fracture	11 (8.9)
Lumbar vertebra fracture	23 (18.5)
**Chest injury**	57 (46.0)
Hemothorax	5 (4.0)
Pneumothorax	21 (16.9)
Pulmonary contusion	27 (21.8)
Rib fracture	34 (27.4)
**Abdominal injury**	20 (16.1)
Solid organ injury	17 (13.7)
Hollow organ injury	2 (1.6)
Retroperitoneal injury	2 (1.6)
**Orthopaedic injury**	63 (50.8)
Pelvic fracture	9 (7.3)
Upper extremity fracture	38 (30.6)
Lower extremity fracture	31 (25.0)

### Comparison of MFTSs

When two scoring systems were compared, both FISS and MFISS scores were significantly higher in males (*p* = 0.029 and 0.026, respectively). Considering the intracranial pathologies in MFT patients, both scores demonstrated significant positive associations with subarachnoidal haemorrhage (SAH) (FISS, *p* = 0.008; MFISS, *p* = 0.027). Additionally, FISS demonstrated significant correlations with cranial fractures and pneumocephalus (both *p* < 0.001), whereas MFISS did not.

Beyond head trauma, FISS values were significantly lower in thoracic trauma patients with pneumothorax (*p* = 0.007) and rib fractures (*p* = 0.004). In contrast, MFISS demonstrated a significant negative association with abdominal trauma (*p* = 0.043). No statistically significant relationships were identified between the two scoring systems in patients with orthopedic injuries. These findings are summarized in Table 3.

**Table 3:** Comparison of FISS and MFISS Scores across injury subgroups

FISS			MFISS	
Injured system	med ± sd (present/absent)	*p*	med ± sd	*p*
Head injury	7.3 ± 4/ 3.8 ± 2.4	**< 0.001**	14.8 ± 13.7/ 10.6 ± 10.4	**0.039**
SDH	7.2 ± 4.9/ 5.3 ± 3,5	0.106	16.5 ± 13.2/ 12.1 ± 12.1	0.088
EDH	7.9 ± 5.6/ 5.3 ± 3.4	0.097	16.1 ± 16.4/ 12.3 ± 11.6	0.300
SAH	7.7 ± 4.3/ 5.2 ± 3.5	**0.008**	18.5 ± 14.3/ 11.5 ± 11.5	**0.027**
Fracture	7.5 ± 3.2/ 4.7 ± 3.7	**< 0.001**	12.5 ± 11.9/ 13 ± 12.6	0.666
Pneumocephalus	8 ± 3.7/ 5.1 ± 3,6	**< 0.001**	12 ± 13.2/ 13 ± 12.2	0.504
Spine injury	4.9 ± 3.3/ 5.9 ± 4	0.190	12.6 ± 11.4/ 12.9 ± 12.7	0.695
Cervical spine	5.1 ± 4.5/ 5.7 ± 3.8	0.391	13.6 ± 11.4/ 12.8 ± 12.4	0.832
Thoracal spine	5.2 ± 3.7/ 5.7 ± 3.8	0.646	9.5 ± 9.6/ 13.2 ± 12.5	0.506
Lumbal spine	5 ± 2.6/ 5.8 ± 4	0.658	14.1 ± 12.6/ 12.5 ± 12.3	0.293
Chest injury	5.1 ± 4/ 6.1 ± 3.6	0.056	13.2 ± 13.4/ 12.5 ± 11.4	0.563
Hemothorax	6.2 ± 5/ 5.6 ± 3.8	0.898	14 ± 15/ 12.8 ± 12.3	0.873
Pneumothorax	4.2 ± 4.3/ 5.9 ± 3.6	**0.007**	9.9 ± 11.5/ 13.4 ± 12.4	0.078
Contusion	5.3 ± 4.2/ 5.7 ± 3.7	0.349	14.8 ± 15.5/ 12.3 ± 11.3	0.903
Rib fracture	4.4 ± 3.8/ 6.1 ± 3.7	**0.004**	12.1 ± 13.6/ 13.1 ± 11.9	0.246
Abdominal injury	4.7 ± 3.1/ 5.8 ± 3.9	0.263	8 ± 9.1/ 13.8 ± 12.7	**0.043**
Solid organ injury	4.5 ± 3.1/ 5.8 ± 3.9	0.219	8.7 ± 9.7/ 13.5 ± 12.6	0.110
Hollow organ injury	6 ± 1.4/ 5.6 ± 3.8	0.618	5 ± 1.4/ 13 ± 12.4	0.518
Retroperitoneal injury	6 ± 5.7/ 5.6 ± 3.8	0.929	4 ± 0/ 13 ± 12.4	0.301
Orthopedic injury	5.9 ± 3.8/ 5.4 ± 9	0.385	14.5 ± 13/ 11 ± 11.4	0.195
Pelvic fracture	5.6 ± 3.4/ 5,7 ± 3.8	0.896	17.7 ± 16.4/ 12.4 ± 11.9	0.308
Upper extremity	6.2 ± 3.8/ 5.4 ± 3.8	0.238	15.6 ± 12.6/ 11.6 ± 12	0.087
Lower extremity	5.7 ± 3.9/ 5.6 ± 3.8	0.968	13.9 ± 14/ 12.5 ± 11.8	0.839

FISS: Facial Injury Severity Score, MFISS: Maxillofacial Injury Severity Score, SDH: subdural hemorrhage; EDH: epidural hemorrhage; SAH: subarachnoid hemorrhage

### Clinical outcomes and prognostic performance of MFTSs

ROC curve analysis demonstrated that neither FISS nor MFISS had significant predictive value for morbidity (AUCs of 0.477 and 0.461, respectively; *p* > 0.05). In contrast, both scoring systems demonstrated strong discriminatory ability for mortality, with AUCs of 0.820 for FISS and 0.758 for MFISS (both *p* < 0.01). A FISS cut-off value of 7.5 yielded a sensitivity of 76.5% and specificity of 82.2% for mortality prediction, whereas an MFISS cut-off of 9 demonstrated a sensitivity of 82.4% with a specificity of 59.8%. These findings indicate that MFTSs may be more informative for identifying patients at risk of death than for predicting overall morbidity in the ED (Table 4).

**Table 4:** Predictive performance of FISS and MFISS for morbidity and mortality in adult multiple trauma patients with maxillofacial injuries presenting to the emergency department

	Morbidity		Mortality	
	FISS	MFISS	FISS	MFISS
p	0.669	0.475	**< 0.001**	**0.001**
Area under curve	0.477	0.461	0.820	0.758
95% Confidence Interval	0.367–0.586	0.352–0.570	0.706–0.934	0.652–0.865
Best cut-off	3.5	17	7.5	9
Sensitivity	66.7%	25.9%	76.5%	82.4%
Specificity	37.2%	74.4%	82.2%	59.8%

**Fig. 1:**
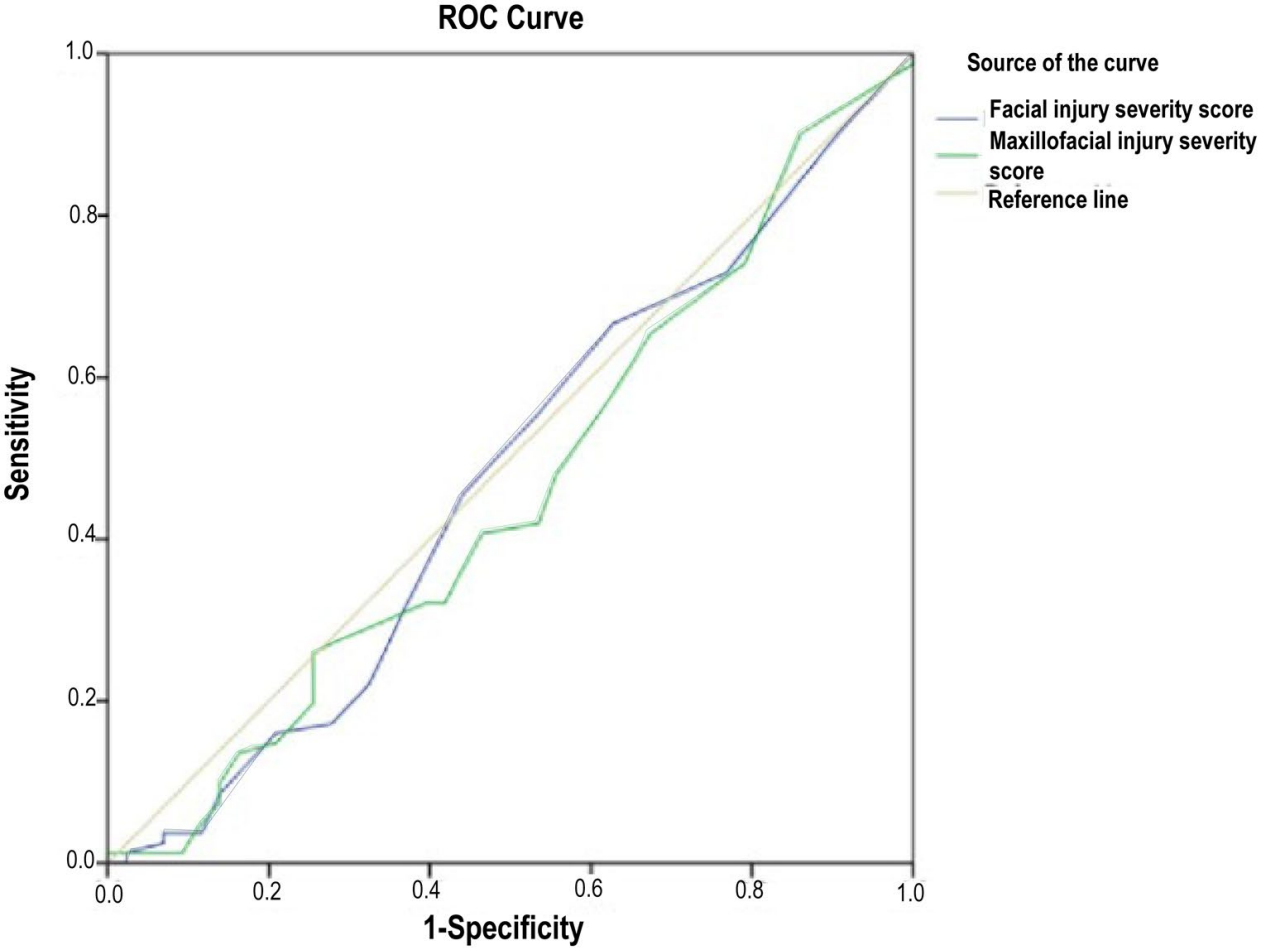
Receiver operating characteristic (ROC) curves demonstrating the predictive performance of FISS and MFISS for morbidity in adult multiple trauma patients with maxillofacial injuries

**Fig. 2:**
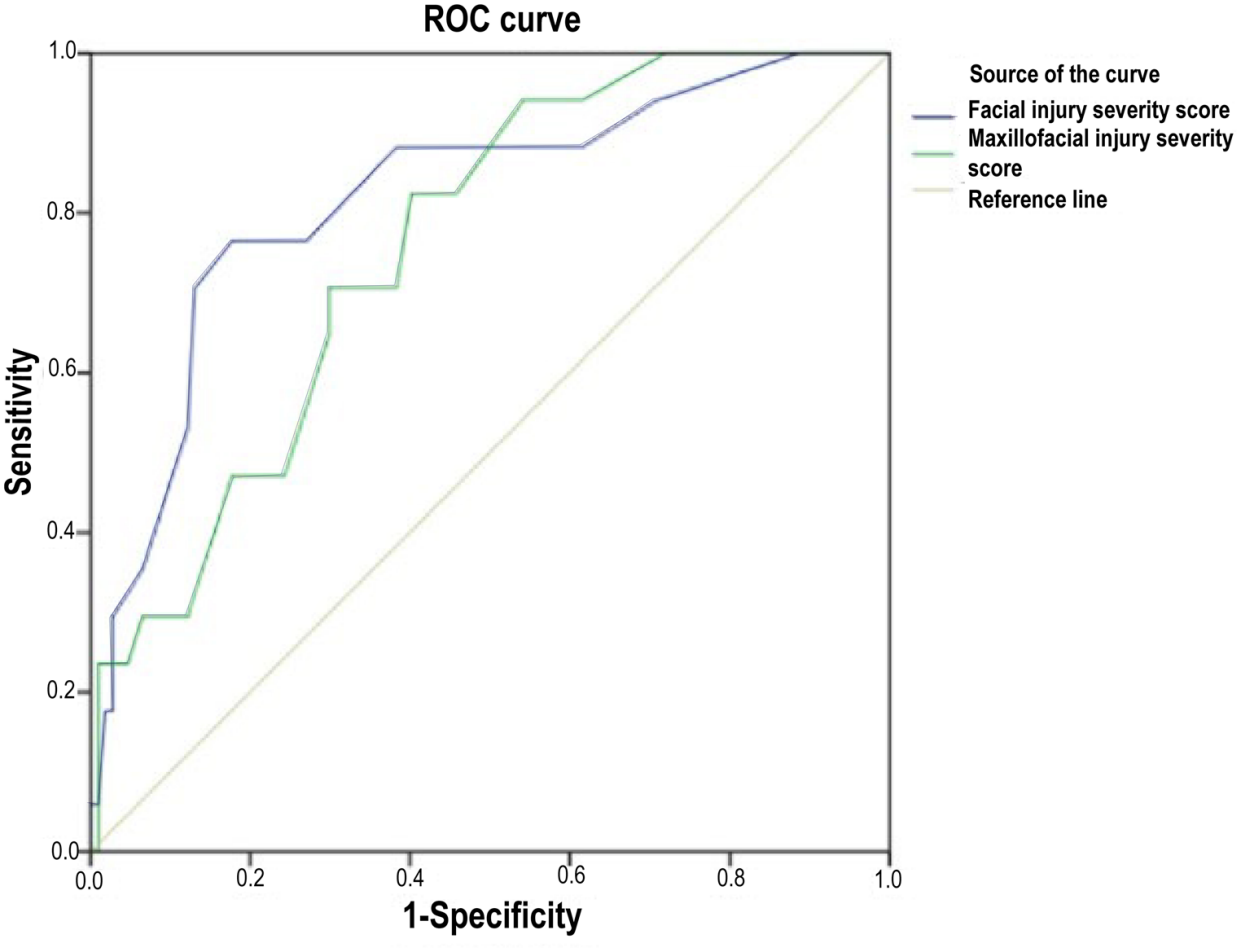
Receiver operating characteristic (ROC) curves demonstrating the predictive performance of FISS and MFISS for mortality in adult multiple trauma patients with maxillofacial injuries

Regarding clinical outcomes, higher FISS scores were significantly associated with intensive care unit admission (*p* = 0.004). Both FISS and MFISS scores were significantly higher in patients who died, indicating a strong association with mortality in the presence of concomitant system injuries (Table 4; Figs. 1 and 2). A total of 108 patients (87.1%) required hospital admission, of whom 57 (46.0%) required ICU admission. The median LOS was 8 days (interquartile range [IQR]: 5–15 days). The mean length of ICU stay was 12.2 ± 12.8 days. Patients with high FISS scores (≥ 7.5) had a longer median hospital stay compared with those with lower scores (10 [IQR: 3–16] vs. 8 [IQR: 5–15] days). In contrast, patients with high MFISS scores (≥ 9) demonstrated a shorter median length of stay than those with lower MFISS scores (7 [IQR: 4–13] vs. 10 [IQR: 5–17] days).

## Discussion

To our knowledge, this study is among the first to directly compare the MFTSs in an ED population of patients with MFT and concomitant injuries. Previous ED-based studies have largely focused on epidemiological patterns, injury mechanisms, or associated systemic injuries rather than the comparative prognostic performance of MFTSs [9–13]. Consequently, early risk stratification and prognostication in this population have remained insufficiently explored. Our findings demonstrate that both FISS and MFISS are associated with clinically relevant outcomes, including ICU admission and mortality, suggesting that these scores reflect not only facial injury severity but also overall clinical risk in the ED (Tables 3 and 4). These findings should be interpreted in the context of contemporary trauma care, where early severity assessment and imaging strategies play a central role in the ED decision-making.

In the ED, where rapid decision-making is required, trauma severity assessment and early imaging strategies are essential components of contemporary trauma care [1, 14, 15]. While trauma algorithms in high-income countries frequently incorporate advanced imaging modalities such as whole-body computed tomography, clinical risk stratification tools play a more prominent role in guiding diagnostic and therapeutic priorities in developing and resource-limited settings [16, 17]. Given the global burden of trauma and the heterogeneity of trauma care infrastructure, simple and reliable severity indicators applicable at the bedside remain crucial across diverse healthcare systems [18–20]. Despite advances in imaging technology, access to such resources continues to vary substantially worldwide.

A potential explanation for this association lies in the biomechanical characteristics of MFT and energy transfer during injury. Because of its physical connection to the base of the skull, the midface can transmit traumatic forces applied to facial structures to the brain. Some facial bones, like the frontal sinus anterior wall, can take a lot of pressure before breaking, but the dura is close enough to the posterior table that kinetic energy can be transmitted to the brain more easily. Therefore, maxillofacial fractures are highly connected with traumatic brain injuries (TBIs) and have been associated with higher rates of morbidity and mortality in cases of head trauma [3, 21, 22]. Consistent with this mechanism, our study demonstrated significant associations between higher FISS scores and specific intracranial pathologies, such as SAH, fracture, and pneumocephalus (*p* = 0.008, < 0.001, and < 0.001, respectively), as well as strong associations with ICU admission. These findings support the concept that fracture burden, as quantified by FISS, may act as a surrogate marker of trauma energy magnitude and neurological risk.

Another noteworthy finding is that FISS was significantly lower in patients with thoracic injuries, such as pneumothorax and rib fractures (*p* = 0.007 and 0.004, respectively). Although it is stated in the literature that the association of MFTs and severe thoracic trauma is common [2], data addressing specific thoracic injury subtypes remain limited. This observation could be explained in part by variations in biomechanical vulnerability. The rib cage exhibits varying structural resilience, influenced by factors such as bone density, cartilage composition, and thoracic protective mechanisms [23]. Furthermore, safety features such as airbag deployment can reduce the force of direct face contact while allowing for thoracic injury; this could lead to a decrease in facial fractures and FISS scores. These findings point to the possibility that injury dynamics, and not injury presence alone, determine the link between the severity of craniofacial trauma and any related injuries.

LOS represents an additional clinically relevant outcome reflecting the downstream course of patients with MFT. Higher FISS scores were associated with longer LOS, although substantial overlap across groups indicated the heterogeneous clinical trajectories typical of polytrauma patients. In contrast, higher MFISS scores were associated with a shorter LOS. This seemingly paradoxical finding may be explained by early clinical decision-making and survivor bias, as patients with significant functional impairment are more likely to undergo rapid escalation of care or experience early mortality, thereby shortening the observed LOS. Collectively, these findings suggest that FISS may better reflect trauma energy and recovery burden, whereas MFISS may capture clinical vulnerability and urgency of management rather than prolonged hospitalization.

Little is known about the correlation between MFISS and injuries outside the craniofacial region. Predicting treatment expenses, LOS, and overall prognosis have been its primary uses in previous research [9]. There was a negative correlation between MFISS and abdominal trauma in our population, but no correlation with thoracic or orthopedic injuries. Injury biomechanics dictate whether force is centered on facial tissues or transmitted to other anatomical regions [6], which may explain why this result contradicts previous literature [8]. It may also represent variations in trauma velocity and impact distribution. Despite these variations, MFISS remained significantly associated with mortality, suggesting that it is more useful as a measure of overall physiological susceptibility than as a marker of specific facial damage.

An important finding of this study is that both FISS and MFISS demonstrated strong predictive performance for mortality but not for overall morbidity, suggesting that MFTSs may be more effective in identifying life-threatening injury patterns rather than the full spectrum of post-traumatic complications. This observation is consistent with previous reports indicating that increasing facial fracture burden reflects higher trauma energy and a greater likelihood of injuries involving other organ systems [7]. From a clinical perspective, these findings support incorporating MFT severity into early ED risk assessment. Rather than serving solely as descriptive measures of facial injury, FISS and MFISS may provide practical insight into global trauma severity and help identify patients who may benefit from closer monitoring, timely intervention, or escalation of care.

### Limitations

When interpreting the findings of this study, several limitations should be acknowledged. First, this was a single-center study conducted at a tertiary care hospital, which may limit the generalizability of the results to other EDs with different patient populations or resource availability. Second, the study population was restricted to patients with MFT accompanied by at least one additional system injury; therefore, the findings may not be applicable to patients with isolated MFT. Third, although the study was prospectively designed, some specific injury subtypes—such as retroperitoneal or hollow-organ injuries—were infrequent, resulting in small subgroup sample sizes and limited statistical power. In addition, prehospital variables, including mode of transportation and prehospital triage characteristics, as well as detailed surgical management data, including the indication for and type of maxillofacial or other surgical interventions, were not systematically recorded and therefore could not be analyzed. Consequently, results related to these less common injury patterns should be interpreted with caution. Fourth, global trauma severity scores, such as the Injury Severity Score (ISS), were not included, which precluded direct comparison between MFTSs and established whole-body injury severity measures. Finally, the study focused on early clinical outcomes, including hospitalization and mortality, and did not assess long-term functional outcomes, morbidity, or quality-of-life measures. Further validation of the clinical utility of MFTSs in the ED will require multicenter studies with larger sample sizes and extended follow-up periods.

## Conclusion

MFT in the ED represents more than an isolated facial injury and may serve as an indicator of overall clinical risk. Our results demonstrate that higher FISS and MFISS scores are significantly associated with serious complications, including intracranial injury, need for ICU admission, and mortality. These findings highlight the potential value of MFTSs as accessible, rapid tools for early risk stratification in the ED, particularly for critically injured patients who require timely clinical decision-making. Integrating such scoring systems into early emergency evaluation may improve the identification of high-risk patients and support prompt, potentially life-saving interventions. However, further multicenter studies are warranted to assess the feasibility, generalizability, and practical implementation of these systems across diverse emergency care settings.

## Data Availability

Data are available from the authors upon reasonable request.
